# Seaweed as a Valuable and Sustainable Resource for Food Packaging Materials

**DOI:** 10.3390/foods13193212

**Published:** 2024-10-09

**Authors:** Aleksandra Nesic, Sladjana Meseldzija, Sergio Benavides, Fabián A. Figueroa, Gustavo Cabrera-Barjas

**Affiliations:** 1 Vinca Institute of Nuclear Sciences—National Institute of the Republic of Serbia, University of Belgrade, Mike Petrovica-Alasa 12–14, 11000 Belgrade, Serbia; sladja_ms@vin.bg.ac.rs; 2 Facultad de Ciencias para el Cuidado de la Salud, Universidad San Sebastián, Campus Las Tres Pascualas, Lientur 1457, Concepción 4080871, Chile; sergio.benavides@uss.cl; 3 Laboratorio de Química de Productos Naturales, Departamento de Botánica, Facultad de Ciencias Naturales y Oceanográficas, Universidad de Concepción, Casilla 160-C, Concepción 4030000, Chile; fabian.figueroa@ucsc.cl; 4 Facultad de Ciencias, Universidad Católica de la Santísima Concepción, Concepción 4060002, Chile

**Keywords:** bioplastic, seaweed films, seaweed packaging, seaweed companies

## Abstract

Plastic food packaging causes massive pollution in the environment via resource extraction, gas emissions, and the enduring plastic waste accumulation. Hence, it is of crucial importance to discover sustainable alternatives in order to protect ecosystems and conserve precious resources. Recently, seaweed has been emerging as a promising sustainable solution to plastic pollution. Seaweed is a fast-growing marine plant that is abundant in tropical coastlines and requires minimal resources to cultivate. In addition, seaweed is rich in valuable polysaccharides such as alginate, fucoidan, carrageenan, agar, and ulva, which can be extracted and processed into biodegradable films, coatings, and wraps. This ability allows the creation of an alternative to plastic food packages that are completely biodegradable, made from renewable resources, and do not linger in landfills or oceans for centuries. In this context, this review discusses the main classification of seaweed, their production and abundance in the world, and provides a summary of seaweed-based materials developed in the last 2–5 years for potential usage in the food packaging sector.

## 1. Introduction

Plastic food packaging presents a major problem on multiple fronts, making it crucial to find replacements. The most significant concern is plastic’s harmful effect on the environment. Traditional plastics are derived from fossil fuels and are not biodegradable. This means they can take hundreds of years to break down, accumulating in landfills and littering landscapes [1]. Even worse, plastic waste often finds its way into oceans, harming marine life through entanglement, ingestion, and the creation of microplastics that pollute the food chain [2]. Improperly disposed plastic packaging can leach harmful chemicals into the soil and water. In addition, some types of plastics, when heated or degraded, can release chemicals that may disrupt hormones or even be carcinogenic [3]. This raises concerns about potential health risks associated with food stored in plastic packaging, especially when heated in microwaves. The production of plastic packaging relies heavily on fossil fuels, a non-renewable resource. This constant demand contributes to resource depletion and the environmental consequences of extracting and refining fossil fuels. While some plastic packaging is recyclable, the recycling process itself can be complex and inefficient. Furthermore, not all types of plastic are readily recyclable, leading to a significant portion ending up in landfills or the environment.

As the world confronts the ever-worsening issue of plastic pollution, seaweed represents immense potential as a sustainable raw source for bioplastics, since it is an abundant and fast-growing marine resource. Unlike traditional plastics derived from fossil fuels, seaweed bioplastics are biodegradable, as they are naturally broken down by microorganisms without leaving behind harmful microplastics that threaten ecosystems [4].

The advantages of seaweed bioplastics extend beyond biodegradability. Namely, specific types of seaweed are rich in polysaccharides that can be extracted and processed into bioplastic films and containers [5,6]. Furthermore, some seaweed species possess inherent antimicrobial properties, which could translate to bioplastics with built-in resistance to bacteria and mold growth [7,8]. In addition, seaweed cultivation requires minimal freshwater and land, making it a low-impact solution compared to traditional agriculture. Moreover, seaweed farms can act as carbon sinks, helping to mitigate climate change.

However, challenges remain in scaling up seaweed bioplastic production. Refining the extraction and processing techniques is crucial to ensure cost-effectiveness and competition with conventional plastics. Numerous studies are being conducted to enhance the mechanical and water vapor barrier properties of seaweed bioplastics to match the durability of their petroleum-based counterparts. Despite these hurdles, the possibilities offered by seaweed for reducing the plastic pollution are undeniable. With continued research and investment, seaweed bioplastics could play a pivotal role in creating a cleaner and more sustainable future.

In light of the above facts, this manuscript aims to provide a comprehensive overview of the last 5 years of seaweed-based food packaging production. It will describe the main parameters that define the quality of seaweed-based materials, their potential and already existing commercial application, and the regulations and safety of these materials for use in the food packaging sector. 

## 2. The Seaweed Revolution

**Figure 1:**
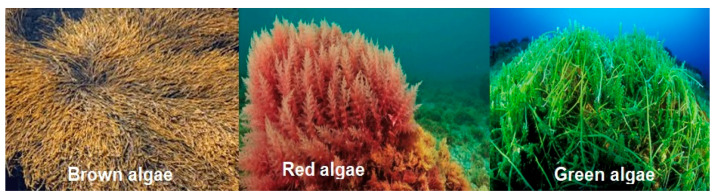
Different types of seaweed.

Seaweed, as a broad spectrum of marine algae, can be classified into three different groups based on their color, cell structure, and pigments (see Figure 1): (a) green seaweed (Chlorophyta), (b) red seaweed (Rhodophyta), and (c) brown seaweed (Phaeophyta). Each group has unique characteristics and offers different benefits [9]. A diverse array of seaweed species, encompassing approximately 900 green, 4000 red, and 1500 brown varieties, exists in nature. Approximately 221 species are currently harvested worldwide, including 32 Chlorophytes, 64 Phaeophyceae, and 125 Rhodophytes [10]. They can contain different distributions of minerals, proteins, and diverse polysaccharides in cell walls, providing beneficial biological properties [11]. The most widely farmed species among three different types of seaweed globally and their chemical composition are displayed in Table 1. As it can be seen from the table, the chemical composition of seaweed varies significantly among the three main types: brown, red, and green. In general, the protein content in seaweed varies between 3 and 45%, and the carbohydrate content varies between 4 and 67%. In addition, seaweed is known to be a material with low lipid content, usually below 8%. Brown seaweed is generally characterized by its high carbohydrate content. Red seaweed typically contains low-to-moderate levels of protein (except in case of Porphyra that contain 45% of proteins) and carbohydrates, with variations depending on the species, whereas green seaweed exhibits higher protein and lipid content compared to brown and red seaweed. These differences in chemical composition are influenced by various factors, including variation in species, environmental, and cultivation conditions.

Due to its high nutritional value, seaweed has been used by humans for centuries for various purposes. In traditional cultures, seaweed has been consumed as food, used in medicinal practices, and employed in industrial applications. Today, the seaweed industry continues to expand, with seaweed-derived products finding their way into a wide range of sectors. Seaweed is used as a food ingredient in Asian culture not only for humans but also as a nutritional feed in aquaculture, providing essential nutrients and unique flavors [19,20]. It is also utilized in the pharmaceutical industry to improve health, due to its recognized high antioxidant and anti-inflammatory properties [21,22,23,24]. Additionally, seaweed-based products have found applications in cosmetics, and agriculture [25,26,27,28,29]. The versatility of seaweed and its growing popularity as a sustainable resource contribute to the continued expansion of the seaweed industry.

In general, all seaweed supply come from two sources: wild harvesting and cultivation (i.e., aquaculture) [30]. Wild harvesting is a traditional method, which involves collecting seaweed directly from natural marine environments. However, overharvesting, or improper harvesting techniques can disrupt marine ecosystems and deplete seaweed populations. Hence, although wild harvesting can be cost-effective, it can also have environmental implications if not managed sustainably [31]. On the other hand, cultivation represents seaweed farming, or mariculture, in controlled environments. This method offers greater control of seaweed growth conditions, such as temperature, light, and nutrient availability [32]. In addition, the cultivation of seaweed in specific locations using sustainable practices can help to reduce pressure on wild populations and ensure a more reliable supply. Additionally, aquaculture can enable the production of specific seaweed varieties with desired characteristics that are tailored to meet market demands.

**Figure 2:**
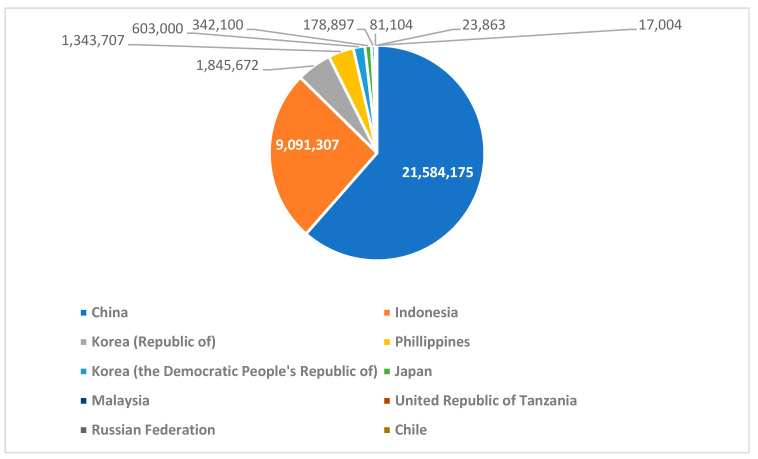
The top ten countries in the world for production of seaweed in tons [33,34].

Currently, almost all seaweed supply comes from aquaculture, cultivated in land-based ponds and near-shore systems. Factors like water temperature, nutrient availability, and light intensity are carefully monitored to optimize growth. Seaweed production offers several benefits, including providing a sustainable source of food, pharmaceuticals, and industrial materials, contributing to coastal economies and promoting biodiversity. According to FAO 2022, 99.5% of farmed seaweed is produced in East and Southeast Asia (35 million tons); China holds the first place with 61% of global seaweed production, and Indonesia holds the second place with 26% [33]. Red seaweed is the most widely cultivated, with a global production of 18,251,474 tons. Brown seaweed is the second most produced, with a global annual production of 16,393,764 tons. Lastly, green seaweed is the least produced, with a global annual production of 73,407 tons [33]. The leading global producers of seaweed are presented in Figure 2. The main exporters of seaweed, according to reports, are Korea (Republic of, USD 254 million), Indonesia (USD 225 million) and Chile (USD 106 million), whereas the main importers of seaweed are China (USD 443 million), the rest of Asia (USD 318 million), and Europe (USD 245 million) [34].

## 3. General Food Packaging Requirements

Food packaging films play a key function in maintaining food quality, safety, and prolonging their shelf life. The selection of the right packaging film depends on the type of food and the various physical–chemical properties of the selected package [35,36]. The most important criteria for food packaging are barrier property and mechanical stability. Barrier property ensures effective resistance against oxygen, moisture, and gases in order to prevent and slow down the spoilage, oxidation, and microbial growth. Additionally, the film must possess sufficient mechanical stability to withstand the transportation and storage time. The industrial standard that food packaging material needs depending on different types of food is presented in Table 2. As it can be seen, food that contains high moisture requires packages with higher moisture and oxygen permeability (MTR and OTR, respectively) when compared to dry food. In fact, the package needs to allow some moisture exchange with the environment to prevent dehydration and the loss of freshness of food with high moisture content. Moreover, fresh food like fruits and vegetables continue to respire after harvest, producing moisture and carbon dioxide. Hence, adequate moisture permeability allows these gases to escape, preventing condensation and the spoilage of the food product. The MTR and OTR values for dry food varies between 0.093 and 30 g/m^2^ day, and 0.068 and 26 cm^3^/m^2^ day bar, respectively. On the other hand, these values for food with high moisture content range between 0.40 and 100 g/m^2^ day and 0.59–10,000 cm^3^/m^2^ day bar, respectively [37]. According to the industrial internal standards, the tensile strength of food packaging materials should be above 7 MPa, and the elongation at break should be above 40% [38]. Besides mechanical stability, the seal ability is also an important parameter to maintain food product integrity and prevent leakage. For products that require visibility, transparency or a clear viewing window is essential. In cases where the product undergoes heating processes, such as cooking or pasteurization, the film must exhibit heat resistance to avoid melting or degradation. Finally, the film should be resistant to chemicals that may come into contact with the food, such as acids or oils.

Food packaging films must comply with regional and international regulations regarding food contact materials. Particularly, the film should not release any harmful substances into the food. This requirement becomes even more important in the case of biobased materials, where the regulations are still not completely clear and implemented. In the last decade, there has been an increased necessity to introduce biodegradable or compostable food packaging materials into the market to reduce plastic accumulation in nature and their negative impact on the environment. Moreover, the film’s production process should consider environmental factors like energy consumption and resource use. Nevertheless, the film should be compatible with existing packaging machinery and processes to minimize production costs [40,41].

## 4. Seaweed-Based Food Packaging

Seaweed has gained increasing attention as a promising raw material for the development of sustainable food packaging due to its numerous advantages. Its renewable nature, biodegradability, edibility, and good oxygen/grease barrier property make it an attractive alternative to traditional plastic packaging [42,43]. However, seaweed is highly sensitive to moisture, which can affect the quality, shelf life, and functionality of seaweed-based materials [44]. To overcome these limitations, researchers are exploring various modification techniques, including blending with other biobased polymers, chemical treatments, and the incorporation of nano-fillers and polyphenols [44,45,46]. These modifications aim to enhance the performance and versatility of seaweed-based packaging in different forms, making it a more viable option for various food products. 

**Figure 3:**
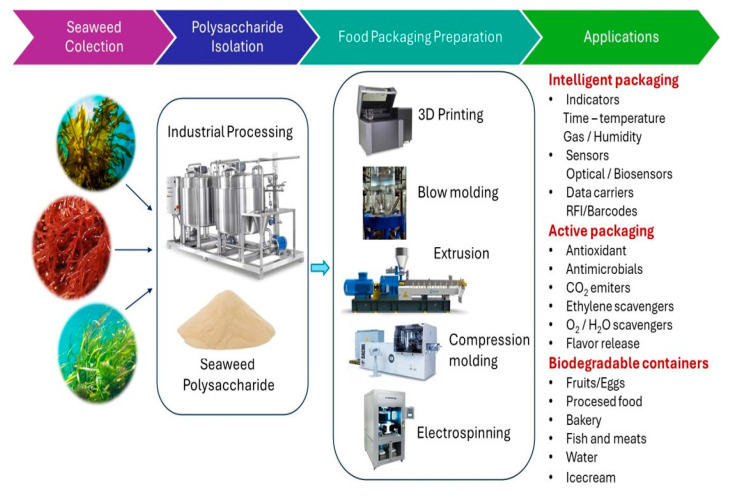
The scheme of processing of seaweed-based food packaging materials.

For example, seaweed pulp can be blended with other biobased polymers and fillers, and processed by extrusion, compression molding, or injection molding to obtain materials for potential use as trays or cups with enhanced strength, flexibility, or biodegradability [47,48]. For specific food products, seaweed may be used to extract valuable polysaccharides that can be further converted into thin films and coatings by 3-d printing, compression molding, electrospinning, or the casting method (see Figure 3). The obtained seaweed-based films can be applied as coatings to fruits, vegetables, and other perishable food to extend their shelf life, reduce moisture loss, and prevent microbial spoilage [49]. Moreover, seaweed-derived coatings can be applied to enhance the properties of paper or other packaging materials due to their high oxygen and grease barrier [50]. These coatings can also be formulated as active food packaging to deliver bioactive compounds, such as antioxidants or antimicrobials, enhancing the nutritional value and safety of the food product [51]. On the other hand, some seaweed-based films can be designed as intelligent packaging by the incorporation of natural functional sensors or pH indicator in order to monitor the quality and freshness of food [52].

Overall, seaweed-based packaging offers a wide range of versatility, allowing for the creation of various packaging formats and applications. The choice of seaweed packaging material depends on the specific product requirements, including its shelf life, and the necessary barrier properties. On the other hand, the final properties of seaweed-packaging materials are strongly influenced by seaweed type and origin [4,44]. Namely, different seaweed species have a varying moisture content, different distribution of polysaccharides, proteins, and bioactive components in cell walls, and different shelf life, which directly impacts the susceptibility to spoilage. Hence, the following sections obtain the summarized data from the literature related to the modifications and physical–chemical properties of food packaging material, classified by the seaweed type and origin.

### 4.1. Brown Seaweed

Brown seaweed is the largest group of seaweeds, known for its large and complex structures like kelp. Their brown color comes from a pigment called fucoxanthin, which masks the green chlorophyll underneath. Brown seaweed is a crucial component of marine ecosystems, providing nourishment and habitat for numerous species. Brown seaweed species typically grows in colder waters. Optimal growth conditions for these algae lie within a temperature range of approximately 20 °C or lower. While brown seaweeds can be encountered in warmer waters, their suitability for valuable polysaccharide production and food applications is often compromised under such conditions. Brown algae is rich in valuable polysaccharides, particularly alginate and fucoidan. These naturally occurring biopolymers act as the building block for brown algae-based food packaging films [53].

#### 4.1.1. Alginate

Alginate is mostly contained in the following genera of seaweed: *Laminaria*, *Sargassum*, *Ascophyllum*, *Macrocystis*, and *Ecklonia*. Alginate is composed of two main building blocks: mannuronic acid (M) and guluronic acid (G). The M and G units are linked together through 1,4-glycosidic bonds. A unique aspect of alginate is its heterogeneity. Unlike some starches or cellulose where the sugar units are all the same, alginate can have varying sequences of MM, GG, and MG units along its chain. This sequence variation (M/G ratio and block structure) can influence the final properties of the alginate. The mannuronic acid are linked through β [1–4] linkage, hence these M-block segments have a linear and flexible conformation, whereas the guluronic acid are linked by α [1–4] linkage, creating a steric hindrance around the carboxyl groups. Alginate has ability to create 3-dimensional gels in the presence of divalent cations. The strength and rigidity of the gel can be controlled by factors like the concentration of alginate and divalent cations, as well as the M/G ratio [54]. For example, a higher concentration of calcium ions and alginate leads to more rigid gels. In addition, it is believed that only G segments interacts with divalent ions in crosslinking reactions [55,56]. Alginate solutions can be cast into thin films upon drying. These films can be flexible or more rigid depending on the processing parameters and the presence of other components [25].

Yun and Liu studied the influence of mandarin peel powder on the physical–chemical properties of alginate-based films. The mandarin peel powder rich in pectin and polyphenols provided high antimicrobial and antioxidant activity to alginate films. The water vapor permeability (WVP) and tensile strength values, depending on type of mandarin peel, were 5.38–8.31 × 10^−11^ g/m s Pa and 4.87–7.90 MPa, respectively. In addition, the obtained films demonstrated a delay in corn oil oxidation [57]. Rahman and Chowdhury developed an alginate–guar gum sensor film for humidity. Namely, the fluorescence intensity of films changed when exposed to a different relative humidity. This ability allowed the monitoring of the freshness of food products like bread [58]. Zhang et al. developed a coating film based on alginate and carboxymethyl chitosan crosslinked with citric acid for the preservation of strawberries. The WVP and tensile strength values of obtained films were 2.74 × 10^−^^2^ g/m day kPa and 1.024 MPa, respectively. In addition, the obtained coating film could preserve the shelf life of strawberries for up to 8 days at 25 °C, 19 days at 5 °C, and 48 days at 0 °C [59].

Capar investigated the influence of vitis vinifera leaf extract and quercetin on the performances of alginate films. It was shown that vitis vinifera leaf extract caused only a slight improvement in the mechanical stability of alginate films (17 MPa) compared to quercetin (16 MPa); however, it had a significant impact on antioxidant activity (51% for alginate–vitis vinifera extract, 36% for alginate-quercetin). All films degraded in soil completely after 30 days [60]. On the other hand, Li et al. incorporated pterostilbene in pectin-alginate matrix. Although pterostilbene improved the antioxidant activity and moisture resistance of films, its addition led to decreased tensile strength [61]. Wang et al. obtained the highest tensile strength (5.3 MPa), antioxidant activity (99%), and water vapor barrier (0.13 × 10^−^^10^ g/m s Pa) with the incorporation of 8 wt% of tea polyphenols in alginate–konjac glucomannan film. Moreover, the film showed high efficiency in the preservation of beef and apples through the inhabitation of the microbial activity, which consequently led to a delay in food spoilage [62]. Akhtar et al. noticed that the addition of 15 wt% of phycocyanin into alginate–carboxymethyl cellulose films caused an enhancement in the water vapor barrier, tensile strength, and ABTS antioxidant activity, reaching values of 5.91 × 10^−^^10^ g/m s Pa, 40.25 MPa, and 80%, respectively [63]. Guo et al. obtained alginate–beetroot extract films incorporated with 10% of carboxylated cellulose nanocrystals, a tensile strength of 55.74 MPa, and ABTS antioxidant activity of 98%. Moreover, the films discolored during the storage of pork when the TVB-N value was above 18.0 mg/100 g, suggesting that the developed film could be efficiently used for the detection of pork spoilage [64]. Santons and Martins demonstrated that adding an onion peel and butterfly pea flower extract inclusion into the alginate matrix led to an enhancement in the mechanical stability by 70% and water vapor barrier by 15% when compared to the control alginate film [65]. Devi et al. studied the influence of onion peel extract on alginate-based films to monitor the freshness of milk. The obtained films had an antioxidant activity of 81% and demonstrated an efficient detection of milk spoilage during storage at 20 °C for 48 h [66].

Aristizabal-Gil et al. demonstrated that the incorporation of ZnO nanoparticles into the alginate matrix at different concentrations up to 5 g/L led to increased WVP from 3.5 × 10^−^^9^ g/m s Pa (control film) to 7.2 × 10^−^^9^ g/m s Pa. This effect was more evident with rising levels of ZnO particles in the film. On the other hand, there was a slight increase in TS, from 78 MPa to 82 MPa, when 0.5 g/L of ZnO nanoparticles was added in the alginate matrix, whereas there was a further increase in the nanoparticle concentration and tensile strength dropped down to 62 MPa. It is interesting to note that ZnO/CaO nanoparticles promoted a better WVP barrier (with incorporation of 5 g/L nanoparticles, WVP = 4.4 × 10^−^^9^ g/m s Pa, but promoted an even higher mechanical destabilization (TS = 54 MPa) [67]. On the other hand, Aziz and Salama investigated alginate-based films incorporated with aloe vera and ZnO nanoparticles. It was shown that the tensile strength increased from 17.1 MPa (control alginate film) to 37.64 MPa (composite film), whereas the WVP decreased from 21.53 mm/m^2^ day kPa (control film) to 6.22 mm/m^2^ day kPa (composite film). Moreover, the composite film demonstrated high antibacterial activity towards *S. aureus* in the efficiency range with commercial synthetic antibiotic ampicillin and towards *E. coli* in the efficiency range of synthetic antibiotic gentamicin. Nevertheless, the coating prolonged the tomato shelf life to 16 days, preventing spoilage [68].

#### 4.1.2. Fucoidan

The most abundant sources of fucoidan include species like *Laminaria*, *Fucus*, and *Undaria pinnatifida*, commonly known as kelp. Fucoidan is characterized by its complex and heterogeneous structure. It is composed of repeating units of fucose, a type of sugar, linked together by glycosidic bonds. The fucose residues can be sulfated at different positions, contributing to the molecule’s overall negative charge. Additionally, fucoidan often contains other sugar residues such as galactose, xylose, and mannose, further increasing its structural complexity. The degree of sulfation, branching, and the presence of other sugars vary depending on the algal species and extraction method, resulting in a diverse range of fucoidan structures [69,70]. Unlike some other polysaccharides like agar or alginate, fucoidan does not exhibit strong gelling properties under typical conditions. The low viscosity of fucoidan and the presence of sulfate groups interfere with the formation of the ordered network structure required for gelation. 

James et al. performed a co-extraction of alginate and fucoidan from Ascophyllum nodosum seaweed in a glycerol/choline chloride solvent, and the co-extract was used for the further development of biobased films. The fucoidan content in films varied between 0 and 100%. The authors obtained an increased WVP and moisture sensitivity values, decreased tensile strength, and increased fucoidan content in films [71]. In addition, Gomaa et al. showed that the presence of fucoidan decreased the water vapor barrier and oxygen barrier of alginate–fucoidan blend films, but improved the antioxidant activity [72]. On the other hand, Wang et al. blended fucoidan with chitosan and added anthocyanins obtained from coleus grass (*Plectranthus scutellarioides*) leaves. The addition of fucoidan in the chitosan matrix led to a reduction in the WVP and OP values, tensile strength, and elongation at break. This effect was further promoted for the WVP, OP, and elongation at break with the inclusion of a higher content of anthocyanin. However, the tensile strength significantly dropped down with anthocyanin presence in the film. On the other hand, the extract combined with fucoidan provided high antimicrobial and antioxidant activity, and was able to detect the spoilage of salmon during the storage time [73]. Liang et al. showed that the inclusion of CA improved mechanical stability, but decreased the water vapor barrier property of chitosan-fucoidan films. These films also had high antibacterial activity, high response to pH, and provided good preservation of litchis during the 8 days of storage [74].

### 4.2. Red Seaweed

Red seaweed is known for its vibrant red color, owing to the phycoerythrin and phycocyanin pigments. These pigments allow red seaweed to absorb blue and green light, enabling them to thrive in deeper waters where sunlight penetration is lower. Red seaweed species are prevalent in colder waters in regions such as Nova Scotia, Canada, and Southern Chile. Additionally, they can be found in more temperate climates, as evidenced by their presence along the coasts of Morocco and Portugal. Furthermore, tropical waters, including Indonesia and the Philippines, also harbor useful red seaweed species. Red seaweed exhibits a diverse range of shapes and sizes, and it has been investigated as a filler for different biobased thermoplastic materials, such as polylactic acid, thermoplastic starch, poly(butylene succinate), and polybutylene adipate terephthalate, produced by reactive extrusion, compression molding, or solvent casting to improve biodegradability and mechanical stability [18,75,76,77]. Additionally, red seaweed is a valuable source of agar and carrageenan, which are used in various food and industrial applications. These complex polysaccharides can be extracted and processed to create bioplastics with unique properties. Carrageenan, for instance, forms gels with varying textures depending on its type, offering versatility in final product design. Agar, known for its gelling and thickening properties, can be mixed with other biopolymers to improve the strength and functionality of films.

#### 4.2.1. Agar

Agar is mostly derived from the genera *Gracilaria* and *Gelidium*. Agar is composed of two molecules: agarose and agaropectin. Agarose is the essential substance that gives agar its gel-forming characteristics. It is a linear polymer composed of D-galactose and 3,6-anhydro-α-L-galactose repeating units linked by 1,3-β-glycosidic and 1,4-α-glycosidic bonds. Agaropectin is a more heterogeneous component of agar. It contains the same basic sugar residues as agarose but also includes additional components such as sulfate and pyruvate groups. The specific ratio of agarose to agaropectin in agar varies depending on the algal species and extraction methods, influencing the final properties of the agar product [78].

Agar is known for its strong gelling properties. Unlike alginate, which requires divalent cations, agar forms gels through a process called thermal gelation. When heated in water, the agar molecules form hydrogen bonds with each other, creating a three-dimensional network that traps water molecules, resulting in a gel. The strength and rigidity of the gel depend on the concentration of agar and the presence of other solutes. One unique feature of agar gels is their thermo-reversibility. Agar gelation involves the transformation of a hot aqueous agar solution into a solid gel upon cooling. This allows for the easy processing and shaping of agar before it sets into a gel upon cooling. The key to this transformation lies in the structure of agar, a polysaccharide composed primarily of agarose. As the solution cools, agarose molecules begin to form a three-dimensional network through the establishment of hydrogen bonds between their constituent sugar units. This intricate network entraps water molecules, resulting in the formation of a firm, translucent gel. The strength and properties of the gel are influenced by factors such as agar concentration, cooling rate, and the presence of other substances [79].

Martinez-Sanz et al. used agar-based extracts from *Gelidium sesquipedale* to obtain the films. The physical–chemical properties of obtained films were compared with ones made by commercial agar. The presence of impurities in agar extract films provoked a higher plasticization effect when compared to commercial agar films. In addition, a longer heat extractive treatment allowed the release of higher amount of polysaccharide, thus providing more ductile, but less resistant films. On the other hand, the WVP values ranged between 0.66 and 0.76 × 10^−^^13^ kg/s m Pa, regardless of the extraction method [80].

Da Rocha et al. studied the influence of fish protein hydrolysate (PH) and clove essential oil (CEO) on the physical–chemical properties of agar films. The tensile strength of both films (agar-PH and agar-CEO films) were lower than for the control agar film. It was interesting to note that the PH inclusion led to an increased elongation at break (42.7%) and solubility (48.86%) of films when compared to the control agar film (elongation at break = 22.4%, and solubility = 21.95%). On the contrary, agar-CEO films demonstrated a significant drop down in the elongation at break (3%) but kept a similar solubility percentage like control films. WVP values increased in both cases, with respect to the control film, and ranged around 3.3–3.7 × 10^−8^ g mm/cm^2^ h Pa. In addition, both films were tested as packages for fish filet, and agar-CEO films provided better fish preservation, which was evidenced by the reduced pH and total volatiles and the slowed-down growth of microorganisms when compared to the control films [81].

Radovanovic et al. developed agar films with the in situ generation of a nano-Cu phosphate and nano-Cu carbonate phase inside the biopolymer matrix. The release of Cu ions into food simulants from both agar-based films over a seven-day period fell within the established specific release limits for this metal. Furthermore, both composite films showed high antibacterial activity towards *E. coli* and *S. aureus* food pathogens, and enhanced water vapor barrier and mechanical stability, when compared to the agar control film. However, Cu-carbonate phase provided films with higher mechanical stability (TS = 39 MPa) and a better water vapor barrier (1.94 × 10^−^^10^ g/m s Pa) than the Cu-phosphate phase [82]. On the other hand, Kumar et al. showed that the incorporation of ZnO nanoparticles into the agar matrix caused a decrease in tensile strength from 51 MPa (control film) to 20 MPa (composite film). Despite lower mechanical stability, composite film preserved the grapes, keeping them fresh up to 21 day stored at room temperature [83]. 

Wang et al. used bacterial nanocellulose from 1 to 10 wt% to improve the physical–chemical properties of agar films. The lowest water solubility (18.55%), lowest WVP (6.88 × 10^−^^11^ g/m s Pa), and highest tensile strength (44.51 MPa) was obtained for agar films that contained 10 wt% of bacterial nanocellulose [84]. Zhao et al. blended agar with different biopolymers such as gelatin, gellan gum, carrageenan; modified it with cellulose nanocrystals as a reinforcement additive; and additionally crosslinked it with calcium ions. The best physical–chemical properties were demonstrated by composite films of agar–gellan gum crosslinked with Ca^2+^ and reinforced with 0.375 mg/g of cellulose nanocrystals. The tensile strength of film was 65 MPa, and the WVP was 5.82 × 10^−^^11^ g/m s Pa. In addition, the composite film preserved the shelf life of strawberries by reducing the weight loss and respiration intensity during 7 days of storage time [85]. 

#### 4.2.2. Carrageenan

Carrageenan is a linear polymer and it is mainly composed of α-D-galactopyranose (Gal), 3,6-anhydro-α-L-galactopyranose (LA), and β-D-galactopyranose 6-sulfate (S-Gal) [86]. The sugar units in carrageenan are linked together by 1,3-β-glycosidic bonds, similar to the main chain linkages in agar. However, the presence of the LA unit can create branches in the carrageenan molecule. The defining characteristic of carrageenan is the presence of sulfate ester groups (SO_3_^−^) attached to the galactose units. These sulfate groups can be located at positions 2, 3, 4, or 6 on the sugar ring, depending on the specific carrageenan type. The number and position of these sulfate groups significantly influence the final properties of the carrageenan. Based on the sulfation pattern (number and position), carrageenan can be broadly classified into three main types: (a) Kappa-carrageenan, which contains one sulfate group per disaccharide unit, is extracted mostly from *Kappaphycus alvarezii* seaweed; (b) Iota-carrageenan, which contains two sulfate groups per disaccharide unit, is mostly extracted from *Eucheuma denticulatum* seaweed; (c) and Lambda-carrageenan, which contains 3 sulfate groups per disaccharide unit, is mostly extracted from *Gigartina* or *Chondrus* seaweed [87]. In addition, 3, 6-anhydrobridges are present in kappa- and iota-carrageenan, but not in lambda-carrageenan.

The presence of sulfate groups allows carrageenan to interact with various cations, affecting its functionality. For example, carrageenan exhibits gelling properties, but the type and strength of the gels depend on the specific carrageenan. Kappa-carrageenan interacts strongly with potassium ions, while iota-carrageenan interacts with calcium ions. Kappa-carrageenan forms strong, rigid gels, whereas iota-carrageenan forms weaker, more elastic gels. Lambda-carrageenan typically does not form gels on its own [88]. Similar to agar, some carrageenan gels (particularly kappa-carrageenan) exhibit thermos-reversibility. They can be melted upon heating and reformed into gels upon cooling. The versatility in gel formation allows for tailoring carrageenan-based bioplastics to specific packaging needs. Currently, due to specific chemical structure, the kappa-carrageenan is the most studied carrageenan. 

Yadav and Chiu developed carrageenan composite films loaded with 1–7 wt% of cellulose nanocrystal, with an increased water contact angle of 72%, enhanced water vapor barrier of 4.69 × 10^−^^11^ g/s m Pa, and improved tensile strength of 53 MPa, in comparison to pristine carrageenan films [89]. Wan Yahaya et al. investigated cellulose nanofibers as a reinforcement additive and butylated hydroxyanisole (BHA) as a strong antioxidant to improve the physical–chemical properties of carrageenan films [90]. The inclusion of cellulose nanofiber increased the tensile strength of film from 35 MPa (control plasticized carrageenan film) to 45 MPa (composite film), but with inclusion of BHA in the composite film from 0.1 to 0.4 wt%, the tensile strength significantly dropped down to 10 MPa. Moreover, the BHA presence up to 0.3 wt% decreased the wettability of carrageenan and carrageenan–cellulose nanofiber films. On the other hand, acorn was used to improve the sealing ability of carrageenan films [91]. The obtained film had a maximum glue and heat synergic sealing strength of 5.09 N/15 mm at 0.3 MPa for 1 s (115 °C). However, the introduction of acorn decreased the water vapor barrier and the thermal and antimicrobial properties of the film. Despite lower physical properties, the film was able to extend the shelf life of beef tallow by significantly minimizing peroxide development within 75 days of storage. In order to decrease the solubility and water vapor transmission of carrageenan films, ZnO and/or SiO_2_ nanoparticles were introduced in the biopolymer matrix and additionally blended with fish gelatin [92]. It was demonstrated that nanoparticles had a synergic effect with fish gelatin on the water-related properties of carrageenan films. The WVP values ranged between 1.34 and 2.67 × 10^−^^10^ g/m s Pa. However, blending composite films with fish gelatin reduced the tensile strength from 28 MPa (carrageenan/nanoparticles) to 17–24 MPa (carrageenan/nanoparticles/fish gelatin). On the other hand, the increased concentration of silver nanoparticles in carrageenan matrix led to a significant drop in opacity from 74% (carrageenan control film) to 1.30% (nanocomposite film) [93]. The tensile strength of obtained nanocomposite films was around 39 MPa, and WVP between 1 × 10^−^^9^ g/m s Pa and exhibited full biodegradation in soil after 4 weeks of exposure. In addition, the films increased the shelf life of strawberries and cheese. 

Avila et al. incorporated jaboticaba extract in the carrageenan matrix to obtain active food packaging films [94]. The inclusion of the jaboticaba extract led to a reduction in the tensile strength value from 7.72 MPa (control carrageenan film) to 3.4 MPa (film with extract) and in water vapor permeability from 1.89 × 10^−^^11^ g/m s Pa (control film) to 1.34 × 10^−^^11^ g/m s Pa. The presence of anthocyanins in the extract caused a color change in films at different pHs, hence demonstrating its ability to be used as an indicator in food packaging films. Liu et al. investigated the freshness of milk by carrageenan films modified with mulberry extract. The incorporation of 4 wt% of extract increased the tensile strength and antioxidant activity of films. The prepared films were able to efficiently detect the deterioration of milk stored at 40 °C during 8 h via the change in film color [95]. On the other hand, Riahi et al. added extracts from sweet potato peel combined with the TiO_2_-doped carbon dots into the carrageenan matrix to monitor the shelf life of shrimps. The obtained films showed 100% antioxidant activity and UV barrier, and high antibacterial activity towards *E coli* and *L. monocytogenes* after only 3 h of incubation [96]. Sangeetha et al. studied carbon dots combined with the coconut husk lignin as a pH color indicator to obtain carrageenan intelligent films for the monitoring of milk freshness [97]. Carbon dots provided good oxygen barrier stability in the films, reaching a value of 83.3 cm^3^/m^2^ day and moderate tensile strength of 35 MPa. The obtained films showed excellent response in an acidic environment via the color change under UV light, thus being able to track the spoilage of milk during this time. Mirzaei et al. monitored the freshness of chicken meat by carrageenan–quercetin and carrageenan/eucalyptus leaf extract [98]. It was shown that eucalyptus leaf extract provided better mechanical stability, antimicrobial efficiency toward food pathogens, and color change at different pH when compared to quercetin, thus giving intelligent film sensors with higher efficiency for chicken spoilage detection. Carrageenan was blended with konjac glucomannan and additionally incorporated with thymol and peppermint oil. The obtained films had high antioxidant and antibacterial activity toward *E. coli*, *L. monocytogenes*, and *S. aureus* due to the synergic effect of the two oils. Moreover, films extended the shelf life of strawberries stored at 4 °C up to 16 days [99]. The introduction of cymbopogon winterianus essential oil into the carrageenan matrix provided films with high antibacterial activity towards *L. monocytogenes*, 100% of antioxidant activity, and a high contact angle (>90 °C) [100]. The tensile strength of films decreased from 46.15 MPa (control nonmodified carrageenan film) to 20.75 MPa (carrageenan–essential oil film).

### 4.3. Green Seaweed

Green seaweed is the most closely related to land plants and shares many similarities in terms of pigments and cell structure. They obtain their green color from chlorophyll, similar to land plants. Green seaweed is particularly abundant in temperate and tropical regions. While brown and red seaweed has gained much attention for their potential use in bioplastics and food packaging sector, green seaweed should not be overlooked. Certain green seaweeds such as Ulva (sea lettuce) show potential for the development of bioplastic films. These bioplastics may offer functionalities like film-forming and gelling properties, paving the way for innovative packaging solutions. In addition, green seaweed biopolymers can be blended with other biopolymers like alginate or carrageenan from brown and red seaweed, respectively. This blending can create composite materials with improved strength, water resistance, and functionality for diverse food packaging applications.

#### Ulvan

Ulvan is mostly extracted from the genera *Ulva*, *Monstroma*, and *Enteromorpha*. Ulvan is an anionic heteropolysaccharide polysaccharide built from repeating units of sugar molecules linked together through 1,4-glycosidic bonds. The primary sugar moieties in ulvan are rhamnose, glucuronic acid, iduronic acid, cellulose, xylose, xyloglucan, glucose, glucuronan, arabinose, mannose, and galactose [101,102]. Generally, ulvan backbone is composed of repetitive sequences of α-L-rhamnose-3-sulfate-1,4-β-D-glucuronic acid and α-L-rhamnose-3-sulfate-1,4-α-D-iduronic acid [103]. 

A defining characteristic of ulvan is the presence of sulfate ester groups (SO_3_^−^) attached to the glucuronic acid and iduronic acid units. The number and position of these sulfate groups can vary depending on the specific Ulva species and environmental factors. This variation in sulfation contributes to the diverse functionalities of ulvan. Additionally, ulvan exhibits excellent film-forming properties [104]. Depending on the degree of sulfation and processing conditions, ulvan can be capable of gelation when exposed to divalent cations. This allows for the creation of hydrogels or more rigid gels, depending on the desired application [105]. In addition, ulvan has been recognized as a chemical with high biological activity due to its specific sugar composition, conformation, content of sulfate groups, low molecular weight, and antioxidant [106,107], anti-tumor [108], immune-stimulating [109], anti-viral activity [110]. 

Guidara et al. investigated the influence of different plasticizers on the physical–chemical properties of ulvan films. In all cases, the water solubility, water vapor permeability (range 1.28–4 × 10^−^^8^ g mm/cm^2^ h Pa), transparency (range from 1 to 7%), elongation at break (range 8–40%), and UV protection of ulvan films increased with an increase in plasticizer content from 1 to 2% *w*/*v*, whereas the tensile strength of films was reduced (range 0.5–3.5 MPa). The ulvan films prepared with glycerol showed higher transparency and mechanical stability than those made with sorbitol [111]. 

Manikandan and Lens showed that ulvan films plasticized with xylitol and citric acid caused growth of gut-friendly microbiota and a reduction in food pathogens such as *E. coli* and *S. aureus*. In addition, the authors demonstrated that the seaweed biomass residues of *Ulva* spp. after ulvan extraction could be used for the efficient synthesis of polyhydroxyalkanoates and the production of films [112]. 

Amin showed that the incorporation of silver nanoparticles into the ulvan matrix had a synergic effect in terms of high antibacterial activity toward several food pathogens. Moreover, the films demonstrated high antioxidant activity with IC50 of 1.128 µg/mL and a moderate-to-high water vapor permeability with a value 1.18 × 10^−^^8^ g mm/cm^2^ h Pa) [113]. Ganesen et al. demonstrated that the presence of carrageenan improved the tensile strength of ulvan films (49 MPa) but led to a higher water vapor permeability (9.96 × 10^−^^8^ g/m s Pa). In addition, they concluded that low-molecular-weight ulvan provided films with higher antioxidant activity, whereas high-molecular-weight ulvan provided films with higher mechanical stability [114]. On the other hand Gomaa et al. showed that ulvan enhanced the UV barrier properties and antioxidant activity of cellulose films, significantly impacted oxygen barrier properties, and also promoted higher water vapor permeation [115]. In addition, Kazemi et al. showed that blending ulvan with gelatin and incorporating a small amount of beeswax (up to 7%) led to a significantly higher water vapor permeability barrier compared to other studies, reaching a value of 1.86 × 10^−^^10^ g/m s Pa. However, the obtained films had a lower tensile strength in comparison to other reported studies (only 6.23 MPa), but high elongation at break (89%) [116].

### 4.4. Comparison of Properties of Seaweed-Based Films from Different Families

Table 3 provides a comprehensive analysis of seaweed-based food packaging materials, depending on the type of seaweed, and highlighting their versatility and potential applications for protection of different type of foods. As it can be seen, brown, red, and green algae were studied to create edible, active, and intelligent films mostly for the protection of fruits, meat, seafood, and dairy products. The data reveal that among the three different types of seaweed, films based on red seaweed have the most consistent WVP barrier, with an order of 10^−^^4^ to 10^−^^6^ g/m day Pa. In the case of brown seaweed-based packages, alginate exhibits a lower WVP barrier (10^−^^2^ to 10^−^^5^ g/m day Pa) than fucoidan (10^−^^5^ to 10^−^^7^ g/m day Pa), whereas the order of the WVP for green algae films is from 10^−^^3^ to 10^−^^6^ g/m day Pa. However, there are not enough fucoidan-based and ulvan-based studies reported in the literature to be able to make further conclusions. Seaweed-based packaging materials demonstrate low-to-moderate WVP barrier properties, regardless of the type of seaweed when compared to commercial food packaging materials. As mentioned, most seaweed-based packages have a water vapor barrier in the range of orders between 10^−^^3^ to 10^−^^5^ g/m day Pa, whereas these values for the common commercial food packaging of dry products are in the order of 10^−^^7^ to 10^−^^9^ g/m day Pa and in some special cases of fruits, 10^−^^5^ to 10^−^^8^ g/m day Pa (see Table 1). Hence, among all the tested types of food, seaweed-based materials might be potentially used only as edible or active coatings for fruits, whereas for meat, oils, and dairy products, they need an improvement in the water vapor barrier by 1 to 5 orders, and for preservation of dry food, they need an improvement by 2 to 5 orders.

Regarding the mechanical stability, the tensile strength, and elongation at break values, seaweed-based films exhibit considerable variation across different formulations, irrespective of the used seaweed species. While the incorporation of nanodots, nanoparticles, and nanocellulose generally enhances the tensile strength of seaweed-based films, ensuring values exceeding 10 MPa, a threshold deemed sufficient for food packaging application, most studied formulations demonstrate relatively low elongation at break, typically ranging between 2 and 30%. In exceptional cases, values as high as 40 to 120% have been observed. In contrast, commercial packages often exhibit significantly higher elongation at break, ranging from 100 to 600%, to protect a wider range of food products. Notably, meat food packaging materials necessitate elongation values between 40 and 100%, while for the powdered food, such as coffee, it is a broader range of 40 to 600% (see Table 1). Furthermore, it is noticed that films produced from brown seaweed possess better elasticity without compromising the mechanical strength, when compared to films made from green and red seaweed. Hence, further research and development are needed to optimize the performance of seaweed-based films, in terms of water vapor barrier and elasticity, to be able expand their applications in the food packaging industry.

### 4.5. Safety of Seaweed-Based Food Packaging Materials

Seaweed-based food packaging materials must comply with safety regulations to ensure the safety of food products and protect consumers’ health. The European Union regulation EU 10/2011 mandates that “food contact materials do not release their constituents into food at levels harmful to human health” [131]. Seaweed, while a valuable natural resource, can pose potential health risks due to its high ability to bioaccumulate certain heavy metals from the aquatic environment. Bioaccumulation occurs when organisms absorb and retain substances from their environment at a faster rate than they can eliminate them. This can lead to the concentration of heavy metals in seaweed, potentially exceeding the safe levels for human consumption [132]. The most common heavy metals accumulated in seaweed are lead, cadmium, mercury, and arsenic. Besides heavy metals, excessive seaweed consumption can cause, in some cases, iodine toxicity. Namely, seaweed is a natural source of iodine and excessive intake can lead to hyperthyroidism, a condition characterized by an overactive thyroid gland [133]. Consequently, food packaging materials derived from seaweed, or its derivatives, may exhibit elevated levels of heavy metals and iodine as well. To mitigate potential health risks, the European Commission established regulations related to the maximum allowed levels of cadmium and lead in different types of food and mercury in edible seaweed; the CEVA organization in France set regulations for lead and cadmium in edible seaweed maximum, and the AFSA organization in France put a limit on iodine in seaweed. These values and references related to the regulations are presented in Table 4. 

Analyzing the seaweed-based formulations presented in previous sections, it can be observed that some formulations contain copper, silver, zinc nanoparticles, or metal salts. According to the European Commission Regulation 10/2011, the maximum allowed migration level of copper and zinc ions from food packaging is 5 mg/kg, whereas for silver ions, it is 10 mg/kg [131]. Hence, the monitoring of content and migration test of heavy metals and iodine from seaweed-based food packaging films into food simulants established by European Commission Regulation 10/2011 is crucial to ensure their safety [131]. Regular testing should be obligatory in order to address any potential issues before products reach the market.

## 5. Commercial Products

**Figure 4:**
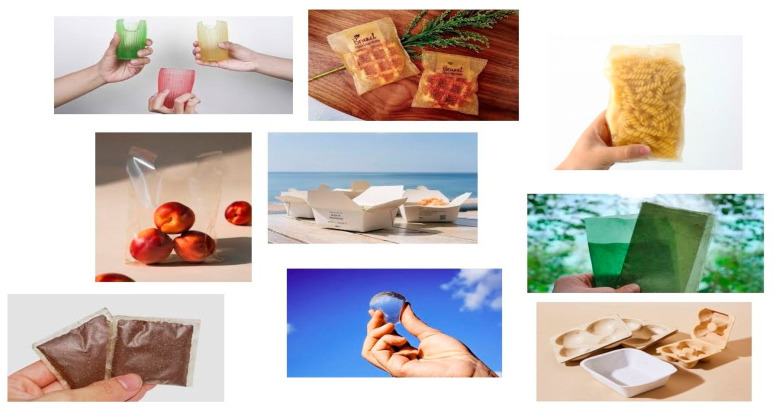
Seaweed-based packaging on the market.

The seaweed packaging industry is rapidly expanding due to its sustainability and eco-friendly benefits. In fact, as consumers increasingly seek sustainable and eco-friendly alternatives to traditional plastic packaging, seaweed-based materials are gaining traction. Seaweed offers a renewable and biodegradable resource that can be transformed into various packaging solutions. Moreover, the unique properties of seaweed, such as its strength, flexibility, and biodegradability, make it an attractive option for packaging applications. The growing interest in seaweed packaging has been leading to increased research and development efforts, and more startups, exploring new technologies and applications for seaweed-based materials. The list of start-up companies that are developing seaweed-based materials for food or the food packaging sector is summarized in Table 5, whereas some of the seaweed-based packaging products currently available on the market are presented in Figure 4. As the industry continues to progress, seaweed packaging is expected to become a more prominent and sustainable solution in the packaging market.

## 6. Conclusions

Seaweed-based food packaging materials offer a promising and sustainable alternative to traditional plastic packaging. Their natural origin, biodegradability, and potential for incorporating functional properties make them attractive options for the food industry. While significant progress has been made in developing seaweed-based packaging by the incorporation of different nanoparticles, nano-fillers, polyphenols, and blending with other biopolymers, further research and development are still necessary to address challenges and optimize their performance. The main parameters for improvement are the elasticity of films, the water vapor barrier, and ensuring compliance with safety regulations. In addition, new food products and packaging formats that can benefit from seaweed-based materials should be explored. By addressing these areas, seaweed-based food packaging can contribute to a more sustainable and circular economy, reducing reliance on fossil fuel-derived plastics and minimizing environmental impact.

## Figures and Tables

**Table 1:** The most widely farmed brown, red and green species in the world [12,13] and their chemical composition [10,14,15,16,17,18].

Seaweed Species	Major Seaweed Producing Countries	Protein, %	Lipid, %	Carbohydrate, %	Fiber, %
**Brown Seaweed**					
***Laminaria***	Japan, Korea, China, Norway, Canada, US, Chile	8–15	1	48	36–37
***Sacharina***	Japan, Korea, China	7–8	1–2	52	10–41
***Undaria***	Japan, Korea, China	12–23	1–5	45–51	16–51
***Sargassum***	China, Philippines	8–16	0.5–1.4	4–68	7–8
***Lessonia***	Chile, Argentina, Peru	13	0.6–1.7	38–48	7–23
***Macrocystis***	US, Canada, Mexico, Chile	11–14	0.3–0.7	42–75	5–18
**Red Seaweed**					
***Kappapchycus***	Philippines, Tanzania, Indonesia, Malaysia, Vietnam	3–7	1	8–65	8–9
***Euchema***	Philippines, Tanzania, Indonesia, Malaysia, Vietnam	5–6	0.2	63–67	6
***Gracilaria***	Chile, Argentina, South Africa, Japan, Indonesia, Philippines, China, India	12	0.3	74	25
***Gellidium***	Japan, China, Korea, Chile South Africa, Portugal, Morocco	9–14	0.1–2	33–40	16
***Porphyra***	Japan, Korea, China	31–44	2	44	12–35
**Green Seaweed**					
***Ulva***	Japan, China, Korea	10–26	0.6–2	36–56	29–55
***Caulerpa***	Indonesia, Australia, Thailand, Philippines, India, France, Spain	12–19	0.9–7.7	49–59	3–12
***Codium***	Australia, Indonesia, Thailand, Philippines, Spain	8–11	0.5–1.5	39–67	5

**Table 2:** Barrier and mechanical requirements of commercial synthetic food packages for different types of food; thickness was approximately between 15 and 25 µm [37,38,39].

Food Product	Shelf Life, Months	MTR, g/m^2^ Day (23 °C, RH = 85%)	OTR, cm^3^/m^2^ Day Bar (23 °C, RH = 75%)	TS, MPa	e, %	Commercial Packages
**Low Moisture Food**						
Nuts, snacks	3–12	0.093–3.0	0.16–9.6	20–80	100–600	Laminates of PP with EVOH, PP; Metallization of PP
Coffee	12–18	0.61–1.1	0.87–1.3	30–80	40–600	PP or PET metallized or AL foil laminates
Other dried foods	12–24	0.14–1.7	0.068–0.82	30–80	100–600	Laminates of PP or PET with EVOH
Oils	>12	<30	2.6–26	45–75	40–600	PET
**High Moisture Food**						
Cheese	2	50	86–345	20–40	100–1000	PP, HDPE
Fat	3	5.2–9.2	6.8–80	30–40	100–600	PP
Retorted food	3–36	0.40–7.6	0.59–5	30–80	100–600	Laminates of PET or PP with EVOH or polyamide
Fruits, vegetables, and salads	0.25	10–4000	10,000–200,000	7–40	200–900	LDPE, PP
Meat and meat-based products	0.25–0.5	2–100	20–10,000	45–75	40–100	PS and PET trays

**Table 3:** The summary of different seaweed-based food packaging materials for different type of food and their water vapor barrier and mechanical stability.

Type of Food Packaging	Film Formulations	Food Product	WVP, g/m Day Pa	TS, MPa	e, %	References
**Brown Seaweed**						
Edible film	**Alginate**–cinnamaldehyde emulsion	Strawberry	5.5 × 10^−2^	62	17	[117]
Edible film	**Alginate**–wheat gluten–soy hull nanocellulose	Banana	1.6 × 10^−4^	60	188	[118]
Edible film	**Alginate**–thymol	Fresh cut apple	4.4 × 10^−3^	28	3	[119]
Edible film	**Alginate**–chitosan–QDs@ZIF-8 nanoparticles	Kiwifruit	3.0 × 10^−3^	30	8	[120]
Edible film	**Alginate**–gelatin–Ag	Tangerine	3.4 × 10^−5^	46	17	[121]
Active film	**Alginate**–aloe vera–ZnO particles	Tomato	6.2 × 10^−3^	38	22	[68]
Edible film	**Alginate**–citric acid.	Cheese	6.8 × 10^−3^	11	20	[122]
Active film	**Alginate**–konjac glucomannan–tea polyphenols	Minced beef	1.7 × 10^−5^	5	80	[62]
Intelligent film	**Alginate**-–cellulose nanocrystals–beetroot extract	Pork	-	55	40	[64]
Intelligent film	**Alginate**–purple onion extract and butterfly pear flower extract	Shrimp and beef	3.7 × 10^−3^	25	5	[65]
Intelligent film	**Fucoidan**–chitosan–coleus grass leaves	Salmon	1.4 × 10^−5^	36	23	[73]
Edible film	**Fucoidan**–chitosan–cinnamaldehyde	Lichi	8.3 × 10^−7^	21	120	[74]
**Red Seaweed**						
Edible film	**Agar**–Zno particles	Green grape	-	29	33	[83]
Edible film	**Agar**–natamycin	Strawberry	3.5 × 10^−5^	15	30	[123]
Edible film	**Agar**–gelatin–carbon dots	Tomato	6.9 × 10^−5^	75	16	[124]
Active film	**Agar**–gelatin–Cu/Zn nanoparticles	Pork	6.1 × 10^−5^	55	12	[125]
Active film	**Agar**–alginate–ginger essential oil	Beef	2.6 × 10^−4^	-	-	[126]
Edible film	**Carrageenan**–peppermint and thymol oil	Strawberry	1.4 × 10^−6^	34	22	[99]
Edible film	**Carrageenan**–sodium carboxymethyl starch	Strawberry	4.8 × 10^−5^	38	27	[127]
Active film	**Carrageenan**–silver particles	Cottage cheese	9.3 × 10^−5^	40	6	[93]
Active film	**Carrageenan**–mulberry extract	Milk	1.2 × 10^−6^	26	9	[95]
Active film	**Carrageenan**–lignin carbon dots	Milk	2.5 × 10^2^	47	22	[97]
Intelligent film	**Carrageenan**–sweet potato peel extract–carbon dots	Shrimp	8.3 × 10^−5^	106	5	[96]
Intelligent film	**Carrageenan**–pectin–sweet potato extract	Salmon	8.1 × 10^−5^	21	24	[128]
Active film	**Carrageenan**–gelatin–date seeds	Goat	1.5 × 10^−5^	9	46	[129]
Active film	**Carrageenan**–honey	Beef	1.4 × 10^−5^	34	79	[130]
**Green Seaweed**						
Edible film	Ulvan–sorbitol	-	3.0 × 10^−6^	2	20	[111]
Edible film	Ulvan–carrageenan	-	8.6 × -10^−3^	49	11	[114]
Active film	Ulvan–gelatin–beeswax	-	1.6 × 10^−3^	6	89	[116]

**Table 4:** Regulations related to the maximum allowed concentrations of harmful substances in food.

Harmful Substance	Maximum Allowed Concentration, mg/kg	Reference
**Cd**	In food: 3	[134]
	In edible seaweed: 0.5	[135]
**Pb**	In food: 0.1–0.5	[136]
	In edible seaweed: 5	[135]
**Hg**	In seaweed: 0.01	[137]
**Iodine**	2000	[138]

**Table 5:** The list of companies that work on development of seaweed-based packages and food materials.

Company	Products
Notpla, UK	Edible liquid pouch, spoons for ice cream, and coatings for cardboard packages
Kelpi, UK	Composite packaging films
FlexSea, UK	Film packages
Solublue, UK	Film packages
PlantSea, UK	Paper, punnets, pods
B’Zeos, Norway	Drinking straws and edible films
Marea, Iceland	Packaging films
Noriware, Switzerland	Biodegradable tableware and food packaging products made from seaweed
Evoware, Indonesia	Edible cups, wraps, films, and sachets
Biopac, Indonesia	Sachet, sheet, pouch, bag
Zerocircle, India	Films, coatings, wood-free paper
Sway, US	Poly and retail bags, resins
Carbonware, US/Puerto Rico	Coatings for cardboard packages and paper
Loliware, US	Drinking straws
ULUU, Australia	Rigid products and fibers

## Data Availability

No new data were created or analyzed in this study. Data sharing is not applicable to this article.
